# A Novel Hybrid Laparoscopic–Extracorporeal Technique for Fertility-Preserving Management of Large Benign Ovarian Cysts: A Case Report

**DOI:** 10.3390/reports9020131

**Published:** 2026-04-25

**Authors:** Sofia Makrydima, Charalampos Milionis

**Affiliations:** “Elena Venizelou” General Hospital, 11521 Athens, Greece; soma20med@icloud.com

**Keywords:** ovarian cysts, fertility preservation, laparoscopy, case report

## Abstract

**Background and Clinical Significance**: The management of large benign ovarian cysts in women of reproductive age requires balancing minimally invasive surgery with oncologic safety and preservation of ovarian function. Laparoscopic cystectomy for large cysts is technically challenging and carries an increased risk of intraoperative rupture and spillage; **Case Presentation**: We describe a novel hybrid laparoscopic–extracorporeal technique in which controlled cyst decompression is performed using a balloon-tipped trocar through a suprapubic port under direct laparoscopic visualization. The ovary is then carefully mobilized and exteriorized through the same incision, allowing extracorporeal cystectomy and ovarian reconstruction before returning the adnexa to the abdominal cavity. This approach was applied in a series of six patients with large benign-appearing ovarian cysts, including one 42-year-old patient with an 18 cm multilocular mature cystic teratoma. There were no intraoperative or postoperative complications, no conversions to laparotomy, and all patients were discharged on postoperative day 1. Follow-up at six weeks and subsequent imaging at nine months demonstrated preserved ovarian architecture, normal menstrual function, and high patient satisfaction; **Conclusions**: The hybrid laparoscopic–extracorporeal approach appears feasible and may offer a safe surgical option in carefully selected patients, allowing fertility preservation while minimizing the risk of spillage. Further studies are needed to evaluate reproducibility, oncologic safety, and long-term reproductive outcomes.

## 1. Introduction and Clinical Significance

The management of large ovarian cysts in young women remains a clinical challenge. Symptoms typically develop once the diameter of the cysts exceeds 10 cm, the threshold at which surgical intervention often becomes necessary. Laparoscopic removal of large ovarian cysts with benign features can be feasible and safe in selected patients [1,2]. In this context, the primary goal is to achieve effective cyst removal while preserving ovarian function and minimizing surgical morbidity. However, as cyst size increases, the risk of intraoperative rupture and spillage rises, largely due to progressive thinning of the cyst wall at sites of maximal distension [3].

Treating apparently benign ovarian cysts with oophorectomy is generally considered undesirable for women of reproductive age because of the adverse impact on fertility. On the other hand, laparoscopic ovarian cystectomy offers the benefits of minimally invasive surgery, including reduced postoperative pain, shorter hospital stay, and faster recovery [4,5]. Although this approach spares fertility, it inevitably carries a small but meaningful risk of unintended cancerous dissemination in the unexpected scenario of an underlying malignancy [6]. Therefore, balancing oncologic safety with fertility preservation necessitates meticulous preoperative evaluation and careful operative planning.

To mitigate the risk of operating on potentially malignant lesions, contemporary practice involves individualized multidisciplinary assessment, at least on an elective basis. This typically includes expert radiologic evaluation, particularly pelvic magnetic resonance imaging (MRI) interpretation, alongside specialist input from gynecologists experienced in both benign and oncologic disease [7]. Only lesions categorized as benign or, at most, borderline based on consensus review are considered appropriate for conservative operative management. However, strategies to minimize the risk of intraoperative dissemination remain essential, even in carefully selected cohorts.

Several methods have been described to achieve controlled cyst decompression and specimen containment during minimally invasive surgery. These include:

1. Mini-laparotomy or small abdominal incision with the application of purse-string sutures or adhesive drapes around the puncture site to allow controlled aspiration and facilitate subsequent cystectomy within a protected field [8].
2. The use of transvaginally introduced laparoscopic bags, placed via a posterior colpotomy into the pouch of Douglas, to collect any inadvertent spillage during dissection [9].
3. Contained in-bag cystectomy, in an effort to conclude resection within an enclosed environment [10].

Despite the obvious advantages of each of these surgical strategies, none of them fully address the combined goals of spillage avoidance, minimally invasive access, and efficient theater time utilization in the setting of very large cysts. In these cases, the increased mobility of the adnexa, facilitated by elongation of the infundibulopelvic ligament due to the cyst weight, presents an anatomical opportunity to refine surgical technique in a way that leverages both open and laparoscopic principles.

In this article, we describe a novel hybrid technique for the safe laparoscopic management of large benign or borderline ovarian cysts. This approach is designed to minimize spillage while maximizing ovarian tissue preservation and reproductive potential. Accordingly, it aims to integrate the benefits of minimally invasive surgery with the controlled environment of open procedures, thereby offering a potential fertility-sparing alternative for selected patients.

## 2. Case Presentation

### 2.1. Patient Information

A 42-year-old nulligravid woman (G0P0) presented with a two-year history of intermittent lower abdominal pain and progressive abdominal distension. She had previously been diagnosed with a large ovarian cyst. However, multiple surgical consultations had recommended open laparotomy, which the patient declined due to her strong desire for fertility preservation and preference for a minimally invasive approach. She had no significant past medical or surgical history, no known gynecologic conditions, and was not receiving any regular medication. Her body mass index was around 24 kg/m^2^. On clinical examination, a palpable abdominopelvic mass extending up to the level of the umbilicus (approximately 18 cm) was noted. Pelvic examination revealed a large, mobile mass occupying the pouch of Douglas.

### 2.2. Diagnostic Assessment

**Figure 1:**
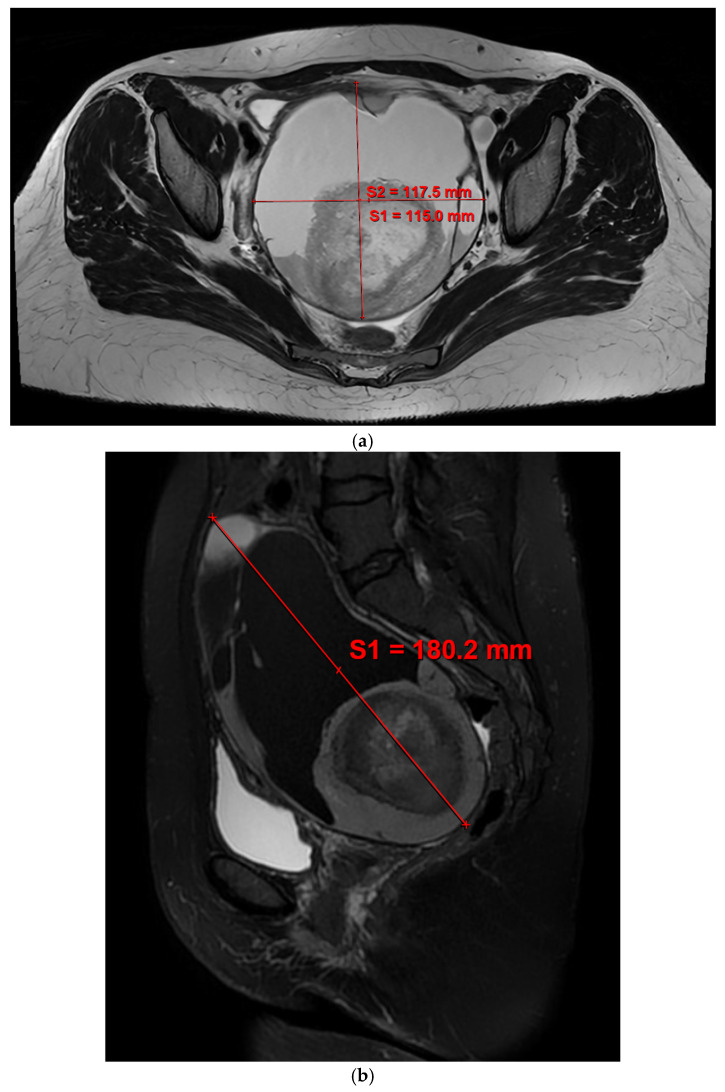
MRI of the ovarian mass: (**a**) transverse view, (**b**) sagittal view.

Transvaginal and transabdominal ultrasound examination was initially performed, revealing a large multilocular cystic mass with echogenic components suggestive of a dermoid cyst. Ultrasound findings were consistent with a benign mature cystic teratoma based on the International Ovarian Tumor Analysis (IOTA) simple rules. Pelvic MRI demonstrated a large (18 cm), multilocular, predominantly cystic adnexal mass with features suggestive, though not pathognomonic, of a mature cystic teratoma, including heterogeneous signal characteristics and areas suspicious for sebaceous content (Figure 1). No solid invasive components or features highly suggestive of malignancy were identified. Serum tumor markers (including CA-125 and CA19-9) were within normal limits. A negative β-hCG value excluded pregnancy. Based on multidisciplinary evaluation, the lesion was classified as likely benign, and the patient was considered an appropriate candidate for fertility-preserving surgical management.

### 2.3. Therapeutic Intervention

The patient underwent a novel hybrid laparoscopic–extracorporeal cystectomy, performed in successive stages.

#### 2.3.1. Patient Positioning, Pneumoperitoneum, and Port Placement

**Figure 2:**
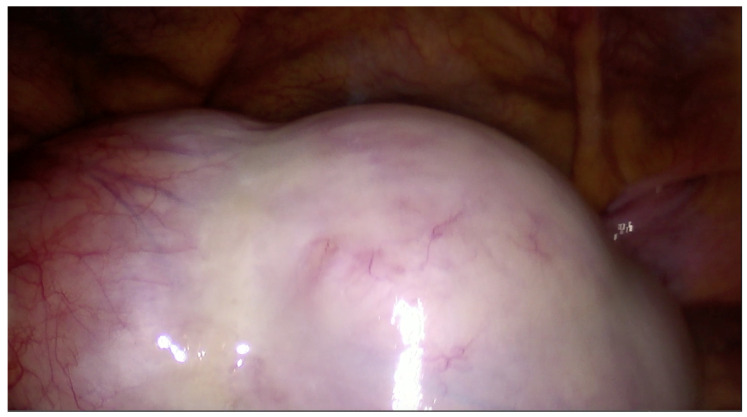
Optimal visualization of a large adnexal mass.

The procedure was performed under general anesthesia with the patient in the dorsal lithotomy position. Pneumoperitoneum was established using the modified Lee–Huang technique, with primary trocar insertion for the laparoscopic camera approximately 3–4 cm above the umbilicus. This entry point minimized the risk of inadvertent cyst puncture while providing an improved panoramic view of large adnexal masses (Figure 2). Two 5 mm ancillary trocars were inserted under direct vision in the right and left iliac fossae to facilitate cyst manipulation and allow adhesiolysis, if required. The Lee–Huang entry point was a preferable alternative to Palmer’s point, as it allows its use as the primary optical trocar, avoiding the need for an additional port. While Palmer’s point is widely accepted, its lateral position may result in suboptimal visualization in cases of large midline masses.

#### 2.3.2. Controlled Cyst Puncture and Decompression

**Figure 3:**
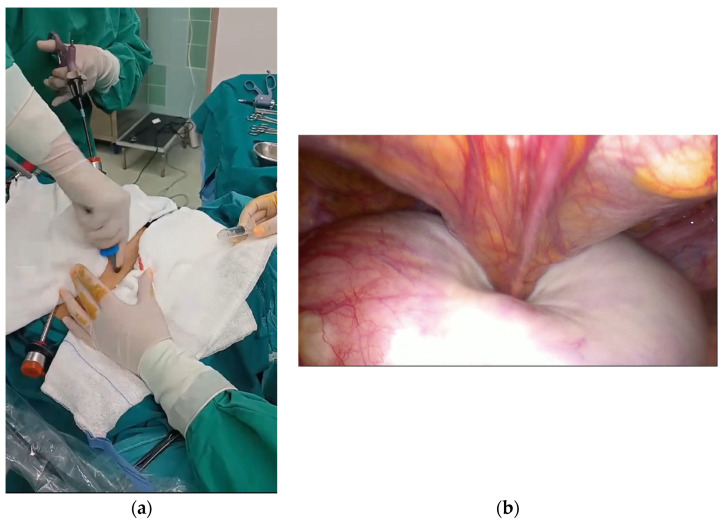
Controlled puncture of the ovarian cyst was performed through the suprapubic balloon-tipped trocar under direct laparoscopic visualization: (**a**) external view, (**b**) laparoscopic view.

**Figure 4:**
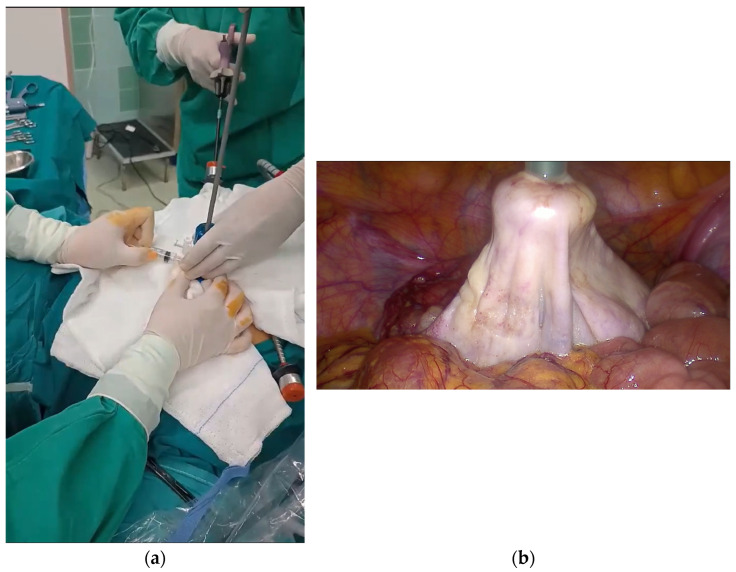
(**a**) Continuous suction was applied via the 10 mm suprapubic trocar (external view). (**b**) Cyst decompression was achieved with minimal intraperitoneal spillage through the effective sealing with the balloon-tipped trocar (laparoscopic view).

A 10 mm balloon-tipped trocar (Kii Advanced Fixation Sleeve; Applied Medical, Rancho Santa Margarita, CA, USA) was introduced through a suprapubic port. Under direct laparoscopic visualization, two atraumatic graspers were used to stabilize the cyst, while controlled puncture was performed through the suprapubic trocar (Figure 3a,b). Simultaneous balloon inflation with gentle traction created a seal between the cyst wall and the trocar, thereby minimizing intraperitoneal spillage. Concurrent suction through the 10 mm cannula allowed rapid and controlled decompression of the cyst (Figure 4a,b).

#### 2.3.3. Management of Multilocular Cysts and Adnexal Mobilization

**Figure 5:**
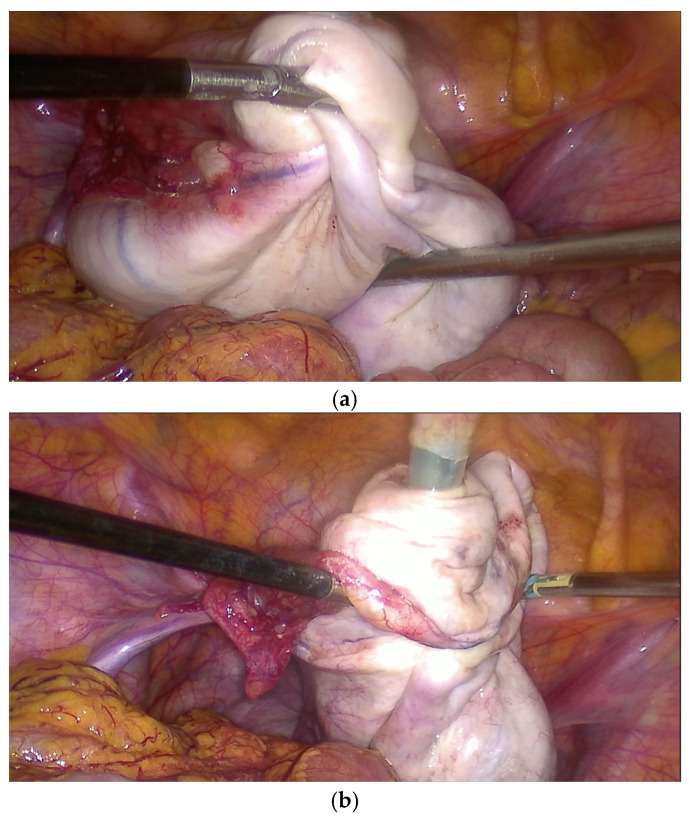
Management of multilocular cysts: (**a**) the trocar was maintained in place while individual locules were sequentially punctured and aspirated; (**b**) mobilization of the adnexa with atraumatic Johan graspers facilitated access to all cyst locules, and the multilocular components were cleared.

The trocar introducer that bore two large holes on its tip was maintained in place. The ovary was then manipulated appropriately with atraumatic Johan graspers to sequentially puncture and suction all locules, thus achieving maximal decompression and improved visualization (Figure 5a,b). In the next step, adhesions between the adnexa and the pelvic sidewall or pouch of Douglas were carefully divided to achieve complete mobilization and facilitate subsequent ovarian exteriorization.

#### 2.3.4. Suprapubic Port Extension and Adnexal Exteriorization

**Figure 6:**
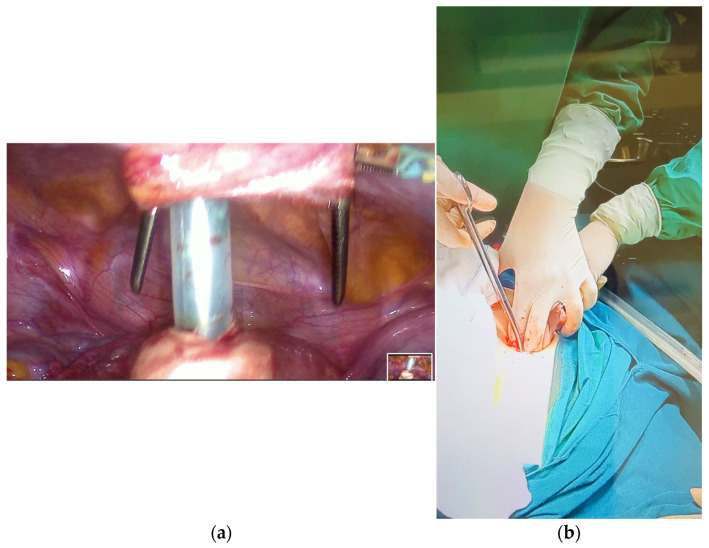
(**a**) Suprapubic port extension using straight atraumatic Pean forceps and scissors under laparoscopic guidance. (**b**) Controlled enlargement of the incision to allow adnexal exteriorization while minimizing trauma to surrounding tissue.

**Figure 7:**
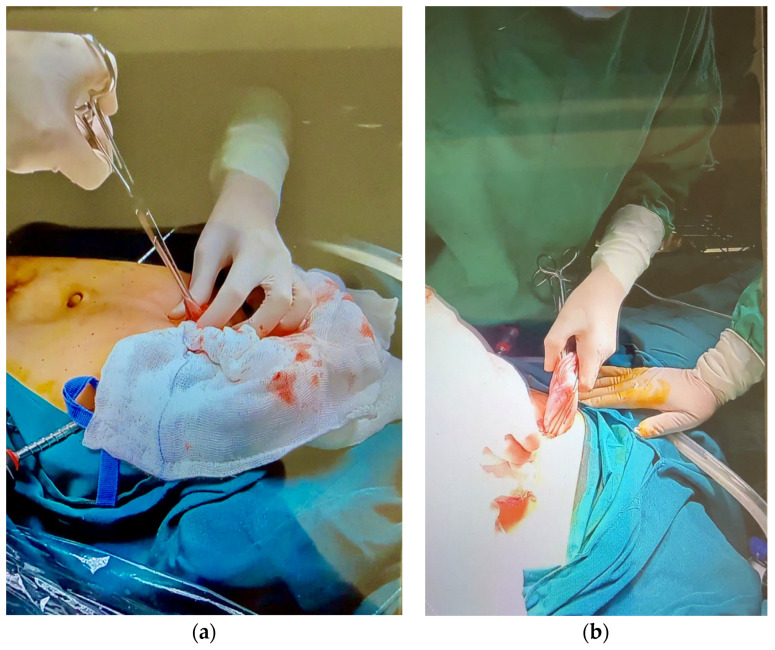
(**a**) Exteriorization of the ovary through the extended suprapubic port for extracorporeal cystectomy. (**b**) Controlled traction during adnexal exteriorization.

The suprapubic incision was then extended under direct vision to approximately 2–3 cm. A straight atraumatic Pean forceps and tissue scissors were used to extend the suprapubic port site as required under direct vision (Figure 6a,b). The balloon trocar was gently delivered through the enlarged incision. Controlled traction was applied to the trocar while laparoscopic guidance was used to mobilize the ovary toward the port site, minimizing the risk of cyst wall tearing due to residual weight. The adnexa were then exteriorized through the abdominal wall (Figure 7a,b). Approximately 2 L of cystic content were aspirated, consisting predominantly of sebaceous material. Solid components including hair, fat, and cartilage were identified and removed.

#### 2.3.5. Extracorporeal Cystectomy and Ovarian Reconstruction

Partial exteriorization of the adnexa was performed. The ovary was incised extracorporeally, and solid components were removed in fragments to allow complete delivery of the adnexa. Extracorporeal ovarian cystectomy was then carried out by carefully separating the cyst wall from healthy ovarian tissue, with minimal use of bipolar energy. Ovarian reconstruction could be performed using interrupted sutures as needed to restore anatomy and prevent hematoma formation.

#### 2.3.6. Repositioning of the Ovary and Peritoneal Washings

Following cystectomy, the ovary was irrigated and returned to the abdominal cavity in its anatomical position. Attention was paid to ensure the absence of torsion of the infundibulopelvic or ovarian ligaments. Peritoneal washings were performed at the conclusion of the procedure to assess for occult spillage in the event of unexpected non-benign final histology.

#### 2.3.7. Optional Modification with In-Bag Controlled Cyst Puncture

Although not performed in this patient, a modification of the technique involves placing the ovary within a laparoscopic specimen retrieval bag prior to controlled trocar puncture as an additional safety measure. This maneuver is relevant and feasible for cysts smaller than 10–15 cm in diameter. When it comes to larger cysts, the risk of cyst rupture during ovarian manipulation outweighs the potential for minimal spillage around the port site.

### 2.4. Follow-Up and Outcomes

**Figure 8:**
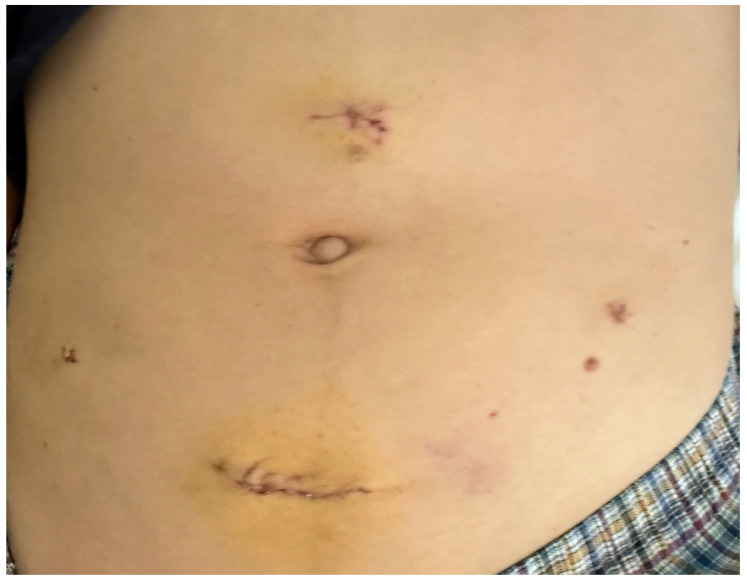
Postoperative cosmetic outcome of the suprapubic incision with minimal scarring and preserved abdominal contour.

**Figure 9:**
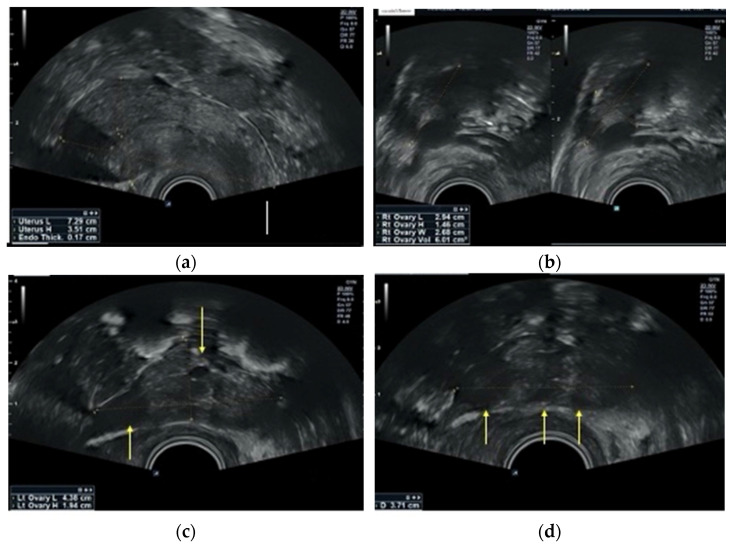
Transvaginal ultrasound on day 3 of the menstrual cycle, 9 months postoperatively. (**a**) Normal-sized anteverted uterus with thin, early proliferative endometrium. (**b**) Right ovary of normal size and homogeneous stromal echotexture. (**c**,**d**) Left ovary (post-cystectomy) demonstrating a typical “collapsed shell” configuration with tubular morphology and heterogeneous echotexture (alternating hypoechoic and echogenic bands). Yellow arrows indicate small cystic foci within the stromal tissue, possibly representing residual antral follicles within the remodeled ovarian parenchyma.

The postoperative course was uneventful, and the patient was discharged the following day. The cosmetic outcome of the technique can be seen in a representative picture of the abdomen on postoperative day 1 (Figure 8). Histopathological examination confirmed a mature cystic teratoma with no evidence of malignancy, while peritoneal washings were negative. At the 6-week follow-up, the patient remained asymptomatic. Transvaginal ultrasound demonstrated normal ovarian morphology without evidence of residual cystic disease, and menstrual function had returned to normal. At 9-month follow-up, transvaginal ultrasound demonstrated preserved ovarian tissue with a heterogeneous echotexture, characterized by alternating hypoechoic bands likely corresponding to fibrotic capsule and stroma, and more echogenic bands representing relatively preserved stromal tissue [11] (Figure 9).

The patient reported a high level of satisfaction with the clinical outcome. She specifically expressed appreciation for avoiding laparotomy and experienced a rapid and comfortable recovery. Notably, she communicated her satisfaction formally to the hospital administration, highlighting the positive impact of the fertility-preserving surgical strategy.

### 2.5. Application of the Technique in a Case Series

This hybrid technique has been applied in five additional patients with large benign-appearing ovarian cysts, forming a total series of six cases. They were operated on by a single surgeon in two tertiary centers between February 2021 and February 2026. All patients were carefully selected following multidisciplinary evaluation, including expert pelvic MRI interpretation and tumor marker assessment, and were considered suitable candidates for fertility-preserving management. The procedures were performed in accordance with institutional standards. Written informed consent was obtained from all patients.

All patients were of reproductive age and presented ovarian cysts larger than 14 cm in diameter. The eventual histopathology revealed three serous cystadenomas and three mature teratomas. The operating time ranged from 80 to 95 min. Estimated blood loss was consistently low across all cases (approximately 50 mL in all cases, based on intraoperative estimation). No intraoperative cyst rupture with macroscopic spillage was observed. There were no intraoperative or postoperative complications, and no conversion to laparotomy was required. All patients were discharged on postoperative day 1. At 6-week follow-up, clinical recovery was uneventful in all cases, and ovaries appeared intact in transvaginal ultrasound. A comprehensive summary of patient characteristics, operative parameters, and postoperative outcomes is presented in Table 1.

## 3. Discussion

The laparoscopic management of large ovarian cysts in women of reproductive age presents a fine balance between minimizing surgical morbidity, preserving ovarian function, and ensuring oncologic safety. The hybrid technique described herein integrates the diagnostic and staging advantages of laparoscopy with the controlled environment of extracorporeal cystectomy. By combining minimally invasive access with extracorporeal ovarian reconstruction, this strategy addresses key limitations of both purely laparoscopic and mini-laparotomy techniques. Therefore, it offers a tailored approach for the management of large benign or borderline ovarian cysts in women seeking fertility preservation.

Unlike previously described laparoscopic-guided mini-laparotomy techniques, the present method uniquely combines balloon-sealed trocar puncture under direct visualization with immediate controlled adnexal exteriorization through the same suprapubic port. Hence, it maintains a temporary closed system during decompression while simultaneously exploiting the increased adnexal mobility induced by cyst weight. This sequence allows controlled extracorporeal cystectomy without the need for an initial abdominal incision or separate containment devices, thereby integrating oncologic vigilance with operative efficiency and fertility preservation.

### 3.1. Initial Assessment of the Peritoneal Cavity

Compared with mini-laparotomy-based approaches [12], the use of the laparoscope allows for an initial comprehensive inspection of the abdominal cavity in accordance with oncologic principles [13]. A thorough assessment of peritoneal surfaces, upper abdominal structures, omentum, contralateral adnexa, and pouch of Douglas takes place prior to cyst manipulation. In contrast to hybrid techniques that commence with mini-laparotomy and subsequently convert to laparoscopy [14], this approach permits real-time modification of the operative plan based on the initial laparoscopic findings. When unexpected metastatic disease is identified, the surgeon may appropriately limit the procedure to biopsies with referral to a tertiary gynecologic oncology center following new counseling and consent of the patient. In case of a profoundly malignant lesion, the surgeon may proceed with a fertility-sparing staging procedure via midline laparotomy, depending on patient consent, surgeon expertise, and institutional resources. The initial laparoscopic view also confirms the feasibility of the procedure and facilitates identification and safe division of possible pelvic adhesions before adnexal exteriorization. Achieving complete ovarian mobilization under direct vision is essential to reduce traction-related injury and to prevent inadvertent ovarian or bowel damage, which could result in cyst rupture, intraperitoneal spillage, or conversion to laparotomy.

### 3.2. Leak-Proof Cyst Decompression

Regardless of the surgical approach employed to treat large ovarian cysts, the initial step involves creating adequate exposure through cyst decompression. Because preoperative differentiation between benign and malignant ovarian masses is inherently probabilistic, dissemination risk must be considered imperative. Realistically, even with meticulous techniques, microspillage can occur during ovarian cystectomies due to oozing around the puncture site as a result of the high intracystic pressure and the fragile cystic walls that may tear unpredictably. There are no follow-up data in correlation to the amount of spillage. However, it can be speculated that microspillage is not visible to the naked eye and hence, it is reported as no spillage in most studies. Meta-analyses have confirmed worse oncologic outcomes for early-stage disease patients who suffered intraoperative spillage [15]. Interestingly, intraoperative spillage has been linked to increased rates of recurrence even for benign cysts and increased rates of chemical peritonitis for dermoid cysts [16].

### 3.3. Extracorporeal Cystectomy

By definition, ovarian cystectomy cannot be executed in a totally contained environment, due to the presence of the infundibulopelvic ligament. Therefore, exteriorization of the ovary followed by extracorporeal cystectomy provides improved control during ovarian tissue manipulation and has been associated with reduced risk of intraperitoneal contamination compared with intracorporeal dissection. Moreover, extracorporeal cystectomy has been shown to significantly reduce operative time compared with fully laparoscopic cystectomy in the context of large ovarian cysts [17]. Shorter operative duration may decrease anesthetic exposure and reduce perioperative morbidity. Routine peritoneal washings performed at the conclusion of the procedure provide an additional oncologic safeguard. In the rare event of unexpected borderline or malignant histology, cytologic evaluation allows reliable assessment of intraperitoneal dissemination, ensuring appropriate postoperative management.

Table 2 presents a detailed comparison of representative surgical techniques as described in the literature.

### 3.4. Considerations in Borderline Tumors and Ethical Implications

The specific technique is primarily intended for benign ovarian cysts. Its theoretical application in carefully selected borderline tumors requires careful consideration. From an oncologic and ethical perspective, the risk of intraoperative spillage, although minimized, cannot be entirely eliminated. Therefore, the use of this approach in such cases should be restricted to highly selected patients following comprehensive multidisciplinary evaluation and thorough informed consent. A thorough preoperative counseling is equally important, ensuring that patients are fully informed about the potential risks, benefits, and uncertainties associated with conservative surgical management.

### 3.5. Impact on Ovarian Reserve

Evidence comparing the impact of surgical approach on ovarian reserve remains limited and heterogeneous. While some studies suggest better postoperative anti-Müllerian hormone (AMH) preservation following laparoscopic surgery, this observation is likely confounded by case selection, as larger and more complex cysts are more commonly treated via laparotomy. Additionally, the abdominal incision itself can negatively influence the ovarian reserve through the formation of postoperative adhesions and associated ovarian trauma. This potential drawback may be attenuated by hybrid surgical approaches. Current data indicate that postoperative ovarian reserve, as assessed by AMH levels and antral follicle count, is primarily influenced by cyst histology, with endometriomas associated with the greatest decline in these indices. Surgical technique also plays a significant role, with an inverse relationship observed between ovarian reserve and the extent of bipolar energy use during cystectomy [23].

### 3.6. Limitations

Nevertheless, this technique has inherent limitations. It should be reserved for carefully selected patients with reassuring preoperative imaging and tumor marker assessment. It also requires advanced laparoscopic expertise with a steep learning curve. Furthermore, its applicability may be limited in cases of dense adhesions, reduced adnexal mobility, or suspicion of invasive malignancy, where en bloc resection or staging laparotomy would be more appropriate. The present technique has been tested in only a small cohort of patients. After all, the incidence of very large ovarian cysts in contemporary practice is relatively low, as most lesions are detected and managed at smaller diameters. Nevertheless, broader adoption should await rigorous evaluation in larger populations. In addition, pre- and postoperative AMH measurements in a cohort of patients would be valuable to better quantify the impact of this technique on ovarian reserve.

## 4. Conclusions

The proposed novel hybrid technique represents a feasible and safe alternative for the minimally invasive management of large ovarian cysts in selected candidates needing fertility preservation. By integrating laparoscopic access with controlled extracorporeal cystectomy, it offers a pragmatic solution to the competing demands of spillage prevention, operative efficiency, and ovarian preservation. Further studies evaluating perioperative outcomes, ovarian reserve, and long-term reproductive results are warranted to better define its role in clinical practice.

## Figures and Tables

**Table 1:** Summary of case series.

Patient	Age (Years)	Cyst Type	Max Diameter (cm)	Operating Time (min)	Blood Loss (mL)	Complications	Hospital Stay (Days)
1	28	SC	14	90	50	None	1
2	23	MT	18	95	50	None	1
3	25	SC	20	85	50	None	1
4	26	SC	16	80	50	None	1
5 *	42	MT	18	90	50	None	1
6	40	MT	20	90	50	None	1

* Index case. The reported blood loss represents an estimate rather than an exact measurement. SC: serous cystadenoma, MT: mature teratoma.

**Table 2:** Existing minimally invasive approaches for the management of large benign ovarian cysts.

Technique	Description	Key Benefits	Key Concerns
Mini laparotomy with leak-proof puncture of ovarian cyst [18,19]	3–5 cm lower abdominal incision with or without self-retaining retractor, adhesive polyethylene bag fixated on ovarian surface, ovary incised to aspirate contents, and progressively extracted from the abdominal cavity	Controlled cyst decompression, extracorporeal ovarian cystectomy	No abdominal assessment, adhesions that may cause inadvertent tears during ovarian mobilization off the abdomen not addressed
Direct cyst puncture followed by contained laparoscopic cystectomy [1]	Direct cyst puncture through a 5 mm suprapubic trocar, decompressed cyst placed in a 5 cm laparoscopic bag	Laparoscopic approach, initial puncture makes laparoscopic cystectomy feasible	No sealing mechanism during initial puncture, laparoscopic instruments need to move within a 5 cm endobag, partial specimen containment
Mini-laparotomy followed by single-port laparoscopic cystectomy [14]	Alexis retractor placed through a mini-laparotomy, direct drainage of the cyst and suturing of the drainage point, sterile glove used to seal the Alexis retractor, fingers of the glove serving as port sites for single-site laparoscopic cystectomy	Controlled cyst decompression, allowing for good laparoscopic views	No initial assessment of the upper abdomen requires advanced technical skills, single port laparoscopic ovarian cystectomy u
Leak- proof aspiration technique and laparoscopic cystectomy [13,20]	Laparoscopic purse-string suture through protective gauze/sterile glove glued on the ovarian wall	Initial laparoscopic assessment, leak-proof during aspiration	Laparoscopic cystectomy technically challenging following cyst aspiration (difficulty in defining planes)
Laparoscopic guided mini laparotomy for benign cysts [21,22]	Laparoscopic aspiration of the cyst and mini laparotomy for cyst exteriorization	Initial laparoscopic assessment, cystectomy after exteriorization	Dissemination risk not considered
Laparoscopic leak- proof cyst decompression and mini laparotomy (this study)	Suprapubic ballooned trocar used for laparoscopic direct cyst puncture, balloon inflated and gently retracted to seal the puncture site, optionally place cyst in a large laparoscopic bag as an additional level of safety when feasible	Leak-proof mechanism during cyst puncture to avoid spillage, extracorporeal cystectomy	Requires a high level of laparoscopic expertise

## Data Availability

The data presented in this study is available on request from the corresponding author. The data is not publicly available due to privacy concerns.

## Abbreviations

The following abbreviations are used in this manuscript:

MRI	Magnetic Resonance Imaging
CA-125	Cancer Antigen 125
CA 19-9	Carbohydrate Antigen 19-9
AFP	Alpha-Fetoprotein
β-hCG	Beta-Human Chorionic Gonadotropin
AMH	Anti-Müllerian Hormone
SC	Serous Cystadenoma
MT	Mature Teratoma
